# Cerebrovascular malformations different from AVMs in patients with hereditary hemorrhagic telangiectasia: a systematic review

**DOI:** 10.1007/s10072-025-08482-3

**Published:** 2025-09-09

**Authors:** Matteo Palermo, Federico Cocilovo, Giuseppe Lanzino, Alessandro Olivi, Carmelo Lucio Sturiale

**Affiliations:** 1 Department of Neurosurgery, Fondazione Policlinico Universitario A. Gemelli IRCCS, Università Cattolica del Sacro Cuore, Rome, Italy; 2 Department of Neurosurgery, Mayo Clinic, 200 First Street SW, Rochester, MN 55905 USA

**Keywords:** Hereditary hemorrhagic telangiectasia, Intracranial aneurysms, Dural arteriovenous fistulas, Developmental venous anomalies, Cavernous malformations, Neurovascular, Cerebrovascular malformations, Systematic review, Genetics

## Abstract

**Background:**

Hereditary Hemorrhagic Telangiectasia (HHT) is an autosomal dominant disorder characterized by abnormal vascular formations across multiple organ systems, including the brain. While arteriovenous malformations (AVMs) are well recognized in HHT, non-AVM cerebrovascular malformations remain underreported and poorly understood manifestations of the disease.

**Methods:**

A systematic review was conducted using multiple databases, applying a two-step screening process to exclude studies with insufficient, irrelevant, or incomplete data. Studies published between 1978 and 2024 were analyzed. The characteristics, clinical presentation, and frequency of non-AVM cerebrovascular malformations, including aneurysms and dural arteriovenous fistulas (dAVFs), were assessed. Pooled prevalence estimates were calculated using a random-effects meta-analysis model.

**Results:**

A total of 1,639 patients with a confirmed diagnosis of HHT were included from 22 studies. The pooled prevalence of non-AVM cerebrovascular malformations was as follows: dural arteriovenous fistulas (dAVFs) 1.2% (95% CI: 0.2–2.2%, *p* = 0.017, I²=85.89%), intracranial aneurysms (IAs) 3.3% (95% CI: 1.5–5.2%, *p* < 0.001, I²=88.68%), developmental venous anomalies (DVAs) 0.2% (95% CI: 0.0–0.5%, *p* = 0.069, I²=0%), cavernous angiomas 0.2% (95% CI: 0.0–0.5%, *p* = 0.058, I²=0%), and capillary vascular malformations (CVMs) 0.4% (95% CI: − 0.1–0.9%, *p* = 0.078, I²=14.34%). Subgroup analysis showed higher IA prevalence in studies lacking systematic screening. Genotype data, when available, suggested ACVRL1 mutations were more common among patients with IAs, while ENG mutations were more frequently associated with brain AVMs and micro-AVMs.

**Conclusion:**

Non-AVM cerebrovascular malformations occur in HHT, with dAVFs showing the strongest association. Other lesions appear sporadic. Genetic subtype may influence lesion type.

## Introduction

Osler-Weber-Rendu syndrome also known as Hereditary Hemorrhagic Telangiectasia (HHT) is an autosomal dominant disorder characterized by abnormal angiogenesis leading to telangiectasias and arteriovenous malformations (AVMs) in multiple organs. The term HHT was introduced in 1909 by the American physician Dr. Hanes, who described its hallmark features [1–8].

Nowadays, HHT is internationally recognized by the diagnostic guidelines provided by the Curaçao criteria: epistaxis, telangiectasias, visceral AVMs, and family history [9].The disorder is associated with mutations of genes involved in the transforming growth factor beta (TGF- β) signaling pathway, which regulate vessel development and homeostasis [10]. Mutations affecting three-key genes have been associated with the onset of the HHT: ENG; ACVRL1, and SMAD4. While SMAD4 mutations lead to a hybrid phenotype of juvenile polyposis and HHT, in the other two cases, manifestations are limited to vascular malformations [7]. Mutations in the RASA1 gene can produce similar vascular features and may sometimes mask the diagnosis of HHT by mimicking telangiectasias [11].

While the association between HHT and nidal AVMs is well established, the relationship between HHT and other cerebrovascular malformations, such as dural arteriovenous fistulas (dAVFs), intracranial aneurysms (IAs), developmental venous anomaly (DVAs), capillary vascular malformations (CVMs), and cavernous angiomas is less well understood, as these lesions are reported less frequently, and their genetic underpinnings remain unclear.

This systematic review and meta-analysis retrieved data from multiple centers to examine the prevalence and clinical findings associated with non-AVM vascular malformations in patients with HHT [12].

## Methods

**Fig. 1:**
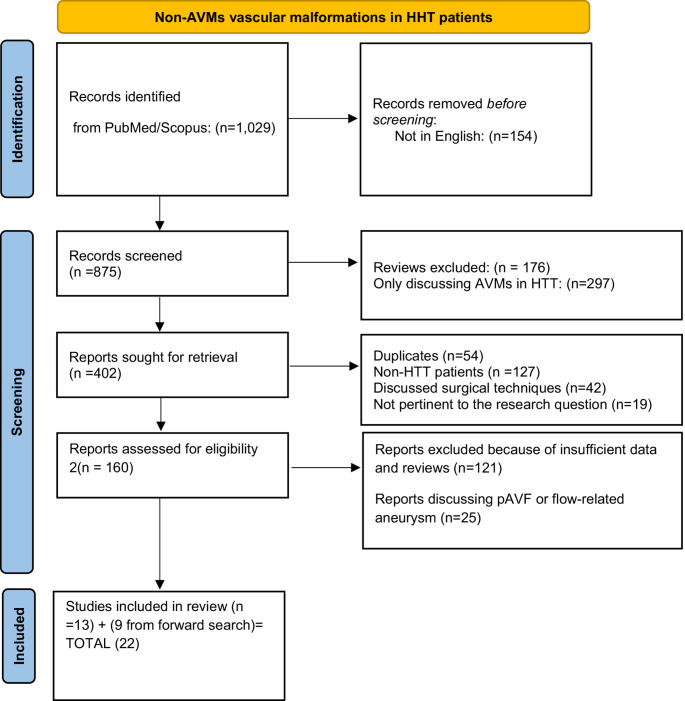
PRISMA 2020 flow diagram for new systematic reviews

This review was performed according to the PRISMA (Preferred Reporting Items for Systematic Reviews and Meta-Analyses) 2020 guidelines [13]. The PICO framework (Population: HHT patients; Intervention: Non-AVMs; Comparison: morphology and managment; Outcome: prevalance) was used to formulate the research question (Fig. 1).

### Search strategy

Two Authors (CLS and MP) performed a comprehensive search on PubMed/MEDLINE and Scopus databases to identify relevant studies discussing cerebrovascular malformations different from AVMs in HHT patients using the search terms: *“(brain OR intracranial OR cerebral OR spinal OR spine OR cord) AND (hereditary hemorrhagic teleangiectasia OR rendu osler OR rendu-osler OR HHT) AND (malformation OR aneurysm OR fistulas OR fistula OR cavernous angioma OR cavernoma OR dural arteriovenous fistula OR AVF OR dAVF OR venous anomaly OR DVA)”*. The search was updated to February 12th 2025, with no time limit. A forward search on references of the retrieved articles was also performed to increase the search power.

### Study selection

The search was restricted to peer-reviewed, English-language studies that included quantitative data. We included studies that reported on cerebrovascular malformations other than AVMs in patients with a genetically confirmed diagnosis of HHT. Animal and pre-clinical studies were excluded, as were review articles and studies lacking explicit data. Two authors (FC and MP) independently screened the titles and abstracts of all articles identified through the search algorithm and selected studies based on the predefined inclusion and exclusion criteria.

After excluding ineligible articles, the full texts of the remaining studies were reviewed to confirm eligibility using the same criteria (Fig. 1). Any disagreements were resolved during a consensus meeting through joint reassessment of the article and the extracted data.

### Data extraction

For each eligible study, we extracted data on the number of patients, mean age, and the number of various cerebrovascular malformations, including aneurysms, DVAs, dAVFs, CVMs, and cavernous angiomas. We distinguished *“flow-related aneurysms”*, located on vessels feeding AVMs, from *“distant aneurysms”*, which were situated on non-feeding vessels, within the Circle of Willis away from an AVM, or in patients without AVMs. Only studies reporting distant aneurysms were included in further analysis. For these, we collected detailed data on patient gender, ethnicity, aneurysm size, location, morphology, rupture status, type of treatment, and both clinical and radiological outcomes. Flow-related aneurysms were excluded from this analysis.

Similarly, for studies describing dAVFs, we extracted information on lesion location, treatment approach, complications, and outcomes at discharge and follow-up. We estimated the prevalence of each malformation type exclusively from studies that reported both the number of genetically confirmed HHT patients examined and the number of malformations detected, thereby excluding isolated case reports that could bias the data. These studies are indicated in Table 1 by the asterisk (*).

**Table 1:** Number of cerebrovascular malformations different from AVMs in patients with genetically confirmed HHT

Author	*N*. of pts with HHT	Mean Age (years)	NO. OF PATIENTS WITH A MALFORMATION				
			Distant Aneurysm	dAVF	DVA	CVM	Cavernoma
**Roman**,** 1978**	2	70.5	1				
**Kamiyama**,** 1981**	1	60	1				
**Fisher**,** 1983**	1	55	1				
**Sobel**,** 1984**	4	46.5					2
**Roy**,** 1990**	1	0.11	1				
**Guillen**, 1991 *	15	NA		1			
**Helmchen**,** 1995**	1	N/A	1	1			
**Gaetani**, 2000 *	77	53.6	3		2		2
**Maher**, 2001 *	321	NA		1			1
**Ling**,** 2005**	1	14		1			
**Chick**,** 2012**	1	60	1				
**Woodall**, 2014 *	209	44	4	1	9	4	2
**Fatania**, 2018 *	59	41	4				
**Alvarez**,** 2020**	1	0.083		1			
**Spangler**,** 2020**	1	32		1			
**Tsang**,** 2020**	1	NR		1			
**Ring NY**, 2021 *	418	41	18				
**Akly**, 2022 *	228	N/A	33				
**Azma**, 2022 *	150	7			31	9	1
**Cheng**,** 2023**	131	34.2	6	1		2	
**Engel**,** 2024**	13	N/A		6			
**Guan**,** 2024**	1	46	1		1		
**TOTALS/+ Stan. Error**	1637	36.87 ± 0.40	75	15	43	15	8

**Legend**: HHT: hereditary hemorrhagic talengectasia; dAVF: dural arteriovenous fistula; CVM: capillary vascular malformation; DVA: developmental venous anomaly

### Statistical analysis

Following the systematic review, we conducted a meta-analysis when multiple studies provided Sufficient data for a given parameter. For each cohort, we calculated the cumulative prevalence and corresponding 95% confidence intervals for each outcome. Outcome rates were then pooled across studies using a random-effects model, which we selected a priori to account for anticipated variability in effect sizes. This model considers both within-study and between-study variance. We assessed heterogeneity among studies using the I-squared (I²) statistic, with values above 50% indicating substantial heterogeneity. All statistical analyses were performed using OpenMetaAnalyst software (http://www.cebm.brown.edu/openmeta/), which is based on R and supported by the Agency for Healthcare Research and Quality (Rockville, MD, USA).

### Risk of bias

**Fig. 2:**
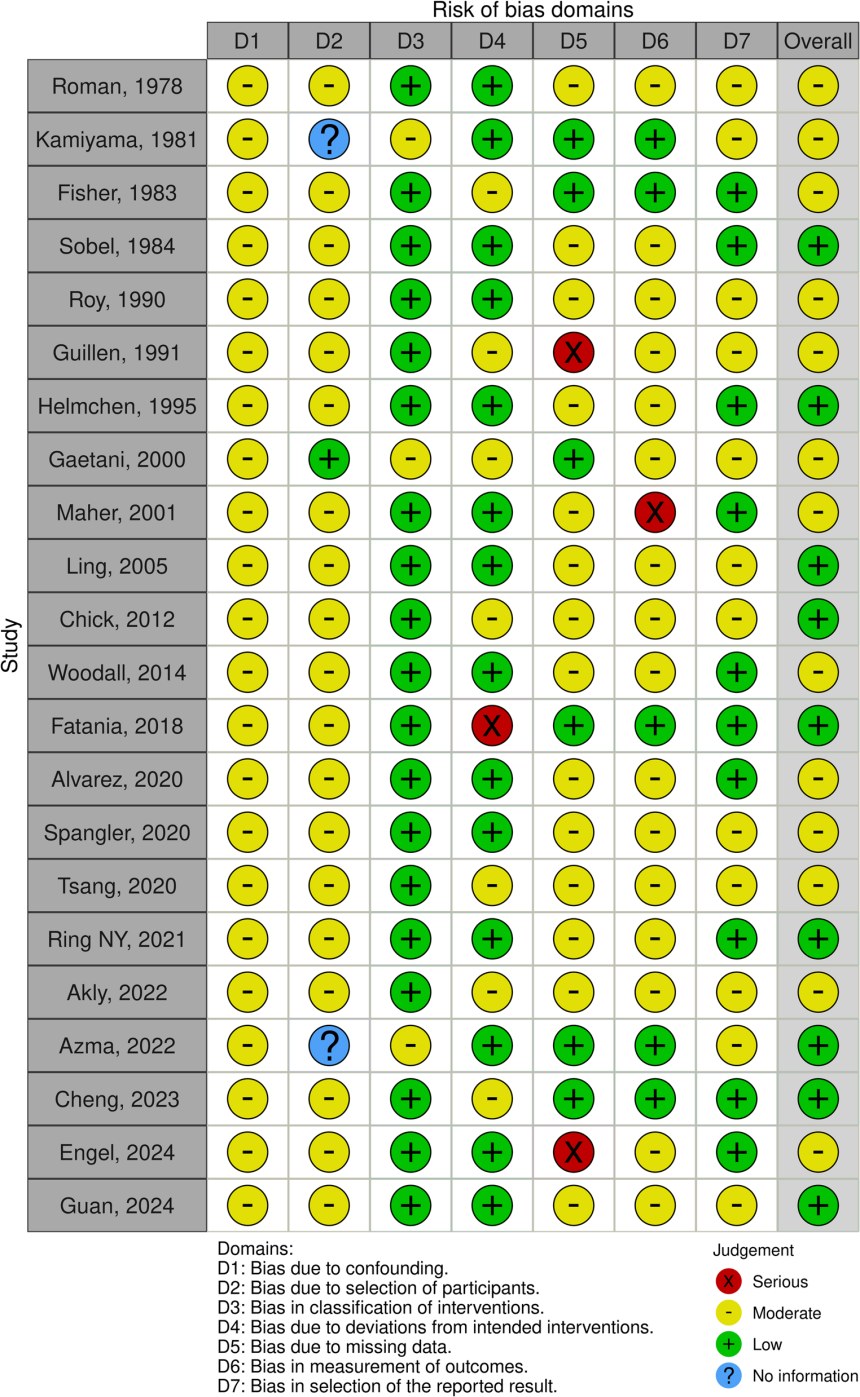
ROBINS-I V2 (Risk of Bias In Non-randomized Studies–of Interventions, Vers. 2)

The ROBINS-I V2 (Risk Of Bias In Non-randomized Studies – of Interventions, Version 2).

assessment tool along with the robins application (https://mcguinlu.shinyapps.io/robvis/) were used to evaluate study quality through visual representations (Fig. 2).

## Results

The search algorithm initially retrieved 1,029 records. During the first screening phase, we excluded non-English publications (*n* = 154), review articles (*n* = 176), studies not addressing AVMs in HHT patients (*n* = 297), duplicates (*n* = 54), papers not involving the target population (*n* = 127), studies focusing primarily on surgical techniques (*n* = 42), and articles deemed irrelevant to the topic (*n* = 19).

The selection phase then focused on identifying studies specifically reporting cerebrovascular malformations other than AVMs in HHT patients (*n* = 160). At this stage, additional exclusions were made due to insufficient or unrelated data and the presence of review articles (*n* = 147). Furthermore, nine articles were identified through forward citation searching and were added to the pool.

Ultimately, 22 studies were included in the final analysis (Fig. 1; Table 1). The quality of these studies was assessed using the ROBINS-I version 2 tool to evaluate the risk of bias (Fig. 2). The study selection process was documented according to the PRISMA 2020 guidelines, with a flow diagram illustrating the phases of identification, screening, eligibility assessment, and final inclusion (Fig. 1).

### Qualitative analysis (Systematic review)

A total of 22 studies published between 1978 and 2024 were included, encompassing 1,639 patients overall (Table 1). The mean age across these studies was 37.87 ± 0.40 years, with patient ages ranging from a few months to 70 years. Most studies included patients who had been pre-screened for cerebrovascular malformations, including both AVMs and other types.

Among the cerebrovascular malformations reported, the most frequently observed were distant aneurysms, identified in 75 patients (4.46%) [4, 8]^14^– [22], followed by 15 dAVFs (0.92%) [17, 21]^23^– [30], 43 DVAs (2.62%), 15 CVMs (0.92%) [14, 17, 21, 31], and 8 cavernous angiomas (0.49%) [14, 17, 25, 31, 32].

### Angioarchitectural and clinical characteristics of dAVFs

Among all patients with HHT included in this review, 15 cases of dAVFs were identified, with spinal involvement being less common (2 cases) than cerebral (5 cases) (Table 2). Of these, 4 patients were treated endovascularly, 2 underwent open Surgery, whereas 1 was managed conservatively with Surveillance, while treatment details were not provided for the remaining 8 cases [14, 17, 21]^23^– [25]^27^– [30, 33].

**Table 2:** Summary of data on HHT patients with dAVF

	DATA STRATIFIED FOR PATIENT WITH DAVF					
Author and year	*N*. pts with dAVFs	Mean age	Location dAVF Spinal/cerebral (S/C)	Treatment	Outcome at discharge	Follow-up
**Engel**,** 2024**	6	N/A	N/A	N/A	N/A	N/A
**Cheng**,** 2023**	1	N/A	N/A	N/A	N/A	N/A
**Tsang**,** 2020**	1	N/A	C (1)	E (1)	N/A	Recurrence dAVF (1)
**Spangler**,** 2020**	1	32	S (1)	E (1)	Monoparesis and Mild UD (1)	Symptom-free (1)
**Alvarez**,** 2020**	1	0.083	C (1)	E (1)	Symptom-free (1)	N/A
**Woodall**,** 2014**	1	N/A	N/A	N/A	N/A	N/A
**Ling**,** 2005**	1	14	S (1)	SU (1)	Symptom-free (1)	Recurrence of dAVF (1)
**Maher**,** 2001**	1	N/A	C (1)	Conservative (1)	Symptom-free (1)	Symptom-free (1)
**Helmchen**,** 1995**	1	N/A	C (1)	E (1)	N/A	N/A
**Guillen**,** 1991**	1	N/A	C (1)	SU (1)	N/A	N/A
**TOTALS**	15	15.36 ± 9.24	C (5); S (2)	SU (2); E(4); Conservative (1)	Symptom-free (3); Monoparesis and Mild UD (1)	Symptom-free (2); Recurrence of dAVF (2)

**Legend**: E: embolization; SU: surgery; DAVF: dural arteriovenous fistula

Outcome at discharge was described in 4 studies. Of these, 3 patients were discharged without symptoms, while 1 had persistent urinary dysfunction (UD) and monoparesis [24, 25, 27, 29]. Follow-up data were available for 4 patients across 4 studies. Two patients experienced recurrence of dAVFs, whereas other two were symptom-free at follow-up [24, 25, 27, 28].

#### Angioarchitectural and clinical characteristics of (distant) intracranial aneurysms

A total of 75 genetically confirmed HHT patients were reported to have 95 aneurysms across 13 studies published between 1978 and 2024 (Table 3). The pooled mean age of these patients was 46.91 ± 6.02 years [4]^16^– [22, 31]^33^– [35]. Ethnicity was explicitly reported in only two patients, both identified as Caucasian [31]. The average aneurysm diameter, available in a Subset of cases, was 8.42 mm [4, 16, 18, 22, 31, 34, 35].

**Table 3:** Summary of data on HHT patients with aneurysm

Authors and year	No. of HHT pts with aneurysm	DATA STRATIFIED FOR PATIENTS WITH ANEURYSM							
		Tot. No. Of Aneurysms	Mean age	Ethnics	Dimension average (mm)	Topography	No. of ruptured	Type of treatment	Radiological outcome
**Roman**,** 1978**	1	1	72	N/A	6	PCom (1)	0	N/A	N/A
**Kamiyama**,** 1981**	1	1	60	N/A	N/A	MCA (1)	N/A	N/A	N/A
**Fisher**,** 1983**	1	2	55	N/A	< 10	ICA (2)	0	Aspirin	Stable size (2)
**Roy**,** 1990**	1	1	0.11	N/A	N/A	N/A	1	SU (1)	N/A
**Helmchen**,** 1995**	1	1	N/A	N/A	N/A	PICA (1)	1	E (1)	Total occlusion (1)
**Gaetani**,** 2000**	3	3	N/A	CA (3)	N/A	N/A	N/A	N/A	N/A
**Chick**,** 2012**	1	3	60	N/A	3.3	ICA (1); MCA (1); PCom (1)	0	N/A	N/A
**Woodall**,** 2014**	4	4	44	N/A	N/A	Opth (1); ICA (2); PCom (1)	0	N/A	N/A
**Fatania**,** 2018**	4	4	N/A	N/A	N/A	ICA (2); Small aneurysm (2)	0	N/A	N/A
**Ring NY**,** 2021**	18	27	N/A	N/A	10.8	ACA (5); B (2); ICA (9); MCA (4); Opth (1); PICA (3); PCA (1); SHA (1); Unspecified (1)	0	Conservative (20); E (3); SU (4)	N/A
**Akly**,** 2022**	33	37	N/A	N/A	12	ICA (9); MCA (9); Opth (8); PCom (3); ACom (2); ACA (1); PCA (2); B (3)	0	N/A	N/A
**Cheng**,** 2023**	6	10	N/A	N/A	N/A	Ant. Circ (10)	0	N/A	N/A
**Guan**,** 2024**	1	1	46	N/A	N/A	PCom (1)	0	E	Total occlusion (1)
**TOTALS**	75	95	46.91 ± 6.02	CA (2)	8.42	ICA (25); MCA (15); Opth (10); PCom (7); ACA (6); B (5); PICA (4); PCA (3); ACom (2); Unspecified small aneurysm (2); SHA (1); Unspecified (1); Ant. Circ (10).	1	SU (5); E (5); Conservative (20); Aspirin (1)	Total occlusion (2); stable size (2)

**Legend**: CA: Caucasian; ICA: Internal Carotid Artery; MCA: Middle Cerebral Artery; Opth: Ophthalmic Artery; PCom: Posterior Communicating Artery; ACA: Anterior Cerebral Artery; B: Basilar Artery; PICA: Posterior Inferior Cerebellar Artery; PCA: Posterior Cerebral Artery; ACom: Anterior Communicating Artery; SHA: Superior Hypophyseal Artery; Ant. Circ: Anterior Circulation; SU: clipping; E: embolization.

Aneurysms were most frequently located in the internal carotid artery (*n* = 25), followed by the middle cerebral artery (*n* = 15), ophthalmic artery (*n* = 10), posterior communicating artery (*n* = 7), anterior cerebral artery (*n* = 6), basilar artery (*n* = 5), posterior inferior cerebellar artery (*n* = 4), posterior cerebral artery (*n* = 3), anterior communicating artery (*n* = 2), and superior hypophyseal artery (*n* = 1). Two cases involved unspecified small aneurysms, and one was reported in the anterior circulation without further anatomical detail [4]^16^– [22, 31]^33^– [35].

Only two aneurysm ruptures were reported in the entire cohort [18, 33]. Regarding treatment, 20 patients were managed conservatively, 5 underwent Surgical clipping, and 5 were treated endovascularly. One patient was treated with aspirin alone [4, 18, 19, 22, 33]. Radiological outcomes were documented in a minority of cases, with two aneurysms reported as totally occluded post-treatment and two demonstrating stable size under observation [4, 22, 33].

### Quantitative analysis (Meta-analysis of prevalence)

**Fig. 3:**
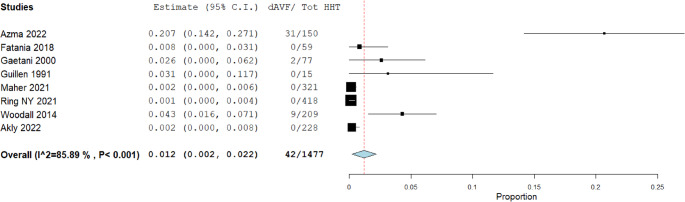
Prevalence of dAVFs in HHT patients

**Fig. 4:**
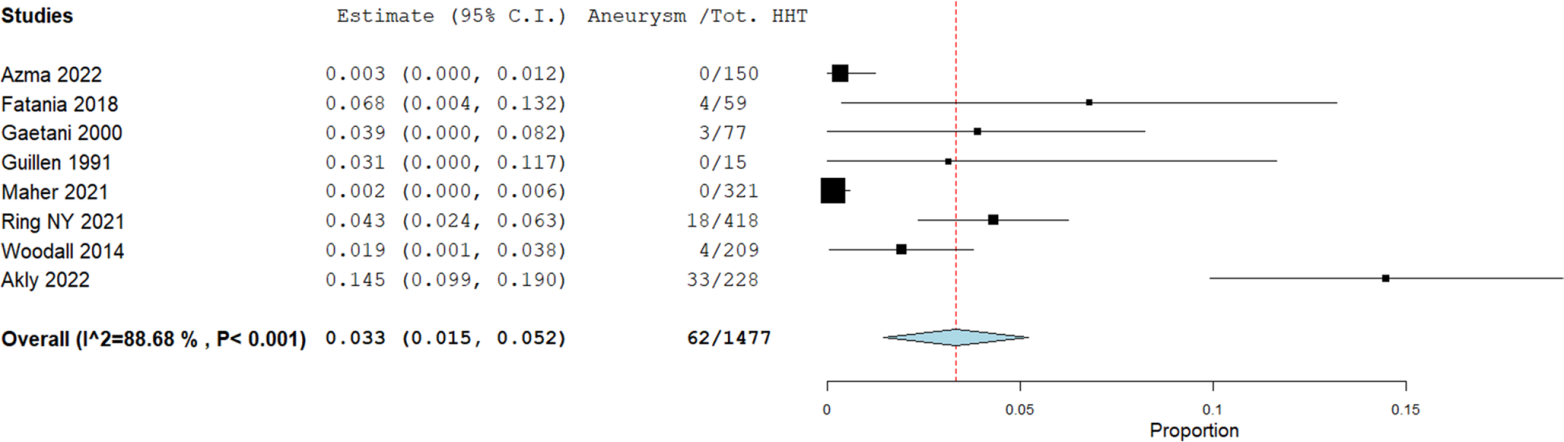
Prevalence of Aneurysm in HHT patients

**Fig. 5:**
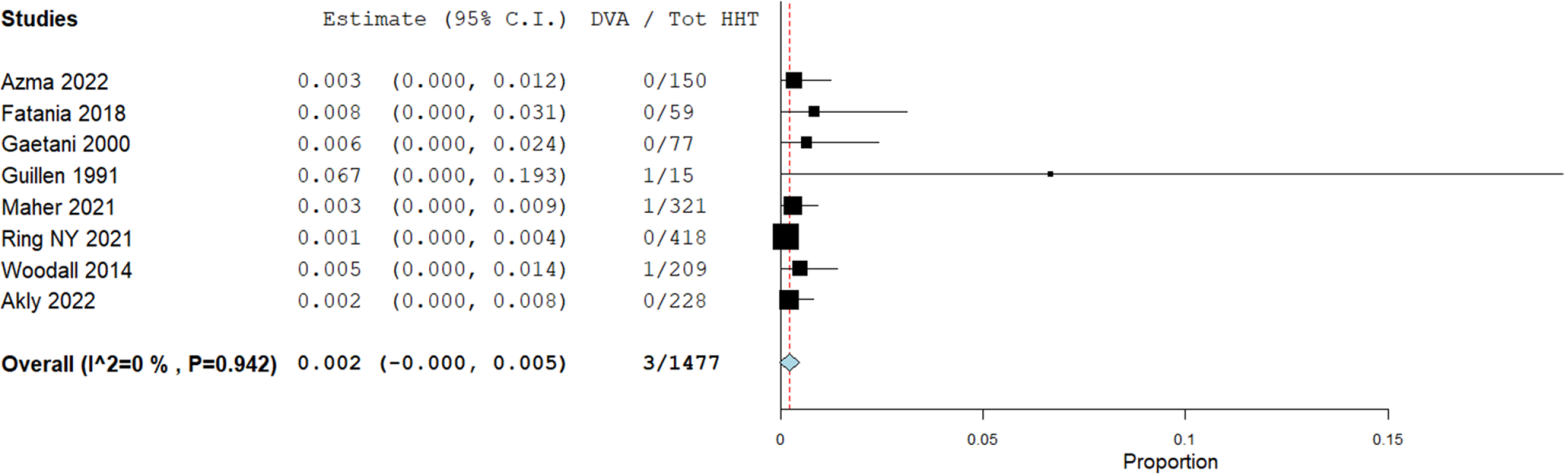
Prevalence of DVA in HHT patients

**Fig. 6:**
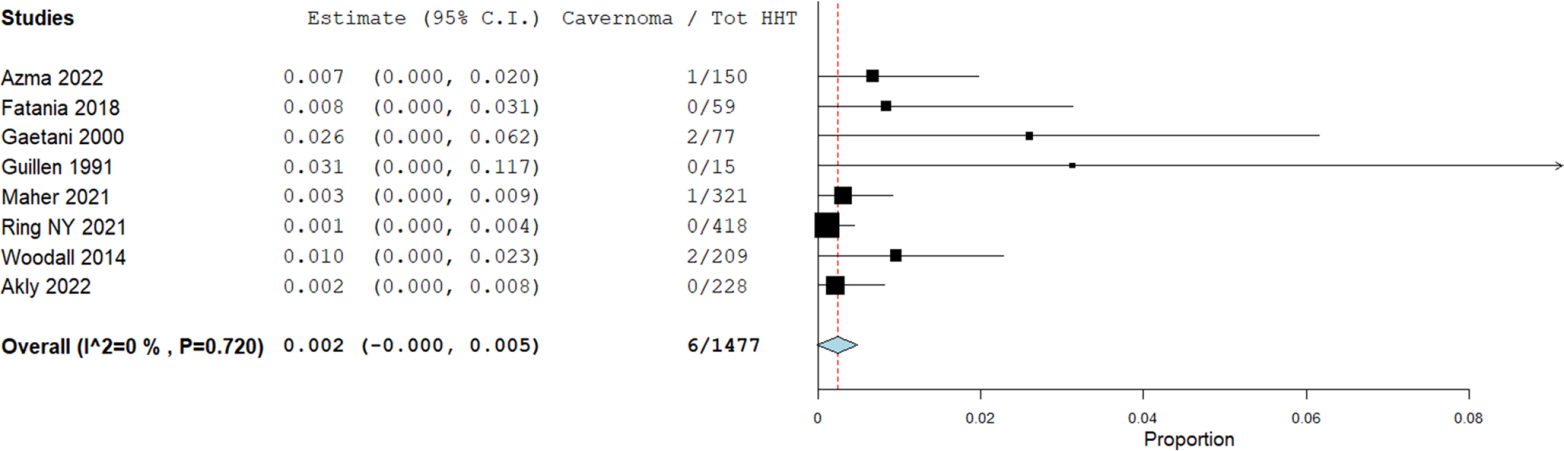
Prevalence of Cavernoma in HHT patients

**Fig. 7:**
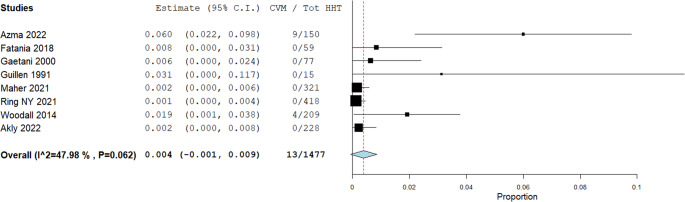
Prevalence of CVMs in HHT patients

Prevalence rates of non-AVMs in patients with HHT are illustrated in Figs. 3, 4, 5, 6 and 7. A total of 1,477 patients were included in the analysis (Table 2). The prevalence of dAVFs was 1.2% (95% CI: 0.002–0.022, *p* = 0.017, I^2^ = 85.89%) (Fig. 3), while distant aneurysms had a prevalence of approximately 3.3% (95% CI: 0.015–0.052, *p* < 0.001, I^2^ = 88.68%) (Fig. 4). DVAs showed a prevalence of 0.2% (95% CI: 0.0-0.005, *p* = 0.069, I^2^ = 0%) (Fig. 5), and cavernous angiomas had a prevalence of 0.2% (95% CI: 0.00-0.005, *p* = 0.058, I^2^ = 0%) (Fig. 6), while the prevalence of CVMs was 0.4% (95% CI: −0.001-0.009, *p* = 0.093, I^2^ = 47.98%) (Figs. 7).

### Study heterogeneity

Heterogeneity in treatment effects across studies was assessed using the I² statistic, with values greater than 50% indicating substantial heterogeneity. Among the prevalence estimates, substantial heterogeneity was observed for IAs dAVFs, reflecting considerable variability across studies. In contrast, DVAs and cavernous angiomas both showed no heterogeneity and CVMs demonstrated moderate heterogeneity, suggesting more consistent findings across studies for these malformation types.

## Discussion

HHT is associated with a wide spectrum of cerebrovascular disorders beyond classical AVMs. A review of available studies examining the prevalence of non-AVMs reveals significant variability in reported rates attributable, at least in part, to the differences in patient selection criteria, cohort characteristics, and methodological approaches. For example, Woodall et al. (2014) analyzed 230 HHT patients, reporting prevalence rates of 4.3% for DVAs, 1.9% for capillary telangiectasias, 1.0% for cavernous malformations, and 2.4% for cerebral aneurysms [17]. In contrast, Ring et al. (2021) and Akly et al. (2022) reported a significantly higher prevalence of aneurysms of 10.3% and 16.3%, respectively [19, 22]. These discrepancies may reflect the exclusion of other malformations in the latter studies and the selective nature of their study populations, and methodological factors such as selection/screening bias, small sample sizes with low event counts in genotype-stratified analyses, confounding by conventional IA risk factors, and underlying biological heterogeneity. These findings underscore the importance of standardized classification and reporting criteria when interpreting prevalence data in HHT populations.

### Non-AVMs cerebrovascular malformations and HHT: possible association or casualty??

Significant insights into potential associations of non-AVM malformations in patients with HHT emerged upon conducting a comparative analysis with the sporadic prevalence of the same malformations in the general population.

The prevalence of dAVFs in HHT patients was 1.2%(Fig. 3), which significantly exceeds the estimated 0.15–0.29% prevalence in the general population [35]. These findings, combined with the angioarchitectural similarities to AVMs, suggest a potential associative link, likely driven by the genetic basis of HHT. However, we acknowledge that our study does not provide definitive evidence of causality. While the prevalence of dAVFs was elevated compared to the general population, the observational nature of the included studies, the limited number of cases, and heterogeneity in screening strategies limit the strength of any inferred association. Additionally, dAVFs are traditionally regarded as acquired lesions, most often associated with venous sinus thrombosis, venous hypertension, trauma, or local inflammation, emerging evidence indicates that a permissive, pro-angiogenic microenvironment is necessary for their development and persistence [36–39]. In HHT, heterozygous loss-of-function mutations in ENG or ACVRL1 disrupt BMP9/10–ALK1 signalling, altering endothelial responsiveness to angiogenic stimuli and promoting endothelial instability with abnormal vascular remodelling. *In agreement*,* a two-step model has been proposed in which* (1) a constitutional predisposition lowers the threshold for aberrant angiogenesis, and (2) a local acquired trigger induces dural neoangiogenesis and pathological arteriovenous shunting, a framework that reconciles the predominantly acquired nature of dAVFs with their apparent higher risk to be developed in HHT patients, thus aligning experimental and clinical evidences implicating both venous hypertension and dysregulated angiogenic signalling in their pathogenesis [36–39]. Therefore, these findings should be interpreted as hypothesis-generating rather than confirmatory.

The association between HHT and IAs remains instead controversial. Brinjikji et al. reported a 2.1% IAs prevalence in HHT patients [3], while Woodall et al. found a similar rate of 2.4% in a cohort of 230 individuals [17]. Ring et al. observed a slightly higher prevalence of 4.3% [19], and a recent study by Perez Akly et al. reported a Substantially elevated rate of 14.5%, nearly three times greater than the estimated 3.2% prevalence in the general population, potentially supporting routine IAs screening in HHT patients [8]. However, our own data revealed a calculated prevalence of 3.3% (Fig. 4), closely aligned with general population estimates (2–5%) [40]. This similarity suggests that HHT may not significantly increase the risk of sporadic (distant) IAs formation, which is more likely driven by conventional cardiovascular risk factors and polygenic predisposition, as previously noted in natural history studies.

A minor discrepancy was noted in the incidence of DVAs, with our study reporting a calculated prevalence of approximately 0.2% (Fig. 5), markedly lower than the 2.6–3.4% prevalence reported in the general population based on autopsy studies [41, 42]. This difference may be attributed to several factors, including underreporting in the original studies due to the incidental and clinically silent nature of DVAs, limited use of contrast-enhanced imaging in older series, and the possibility that DVAs are underrecognized or not systematically sought in HHT patients due to the diagnostic focus on high-flow vascular lesions such as AVMs and dAVFs.

Similarly, the prevalence of CVMs in HHT patients in our cohort was 0.4% (Fig. 7), aligning closely with general population estimates (0.4–0.9%) [43]. The same was true for cavernous angiomas, with a prevalence of 0.2% in our study compared to 0.4–0.8% reported in the general population (Fig. 6) [44].

### Genetics of non-AVM vascular malformations

Genetic subtypes play a pivotal role in shaping the phenotypic spectrum of cerebrovascular manifestations in HHT.

For instance, both Woodall et al. and Ring et al. reported that the majority of HHT patients with IAs had mutations in ACVRL1 gene (HHT2) [45]. Similarly, Saliou et al. identified distinct genotype–phenotype correlations in pediatric cases, noting that ENG mutations, associated with HHT1, were primarily linked to brain CVMs and micro-AVMs. In contrast, RASA1 mutations, associated with CM-AVM syndrome, were more commonly linked to multiple micro-AVMs, including vein of Galen malformations (vGaMs), as well as cutaneous capillary anomalies. This comparison highlights the differing cerebrovascular profiles between RASA1 syndrome and HHT [46]. In the study by Akly et al., genetic data was available for 81 of 228 HHT patients (35.5%), of whom 9 had IAs. Among these, 4 had ENG mutations, 3 had ACVRL1 mutations, and 2 were inconclusive; no SMAD4 mutations were detected. However, no statistically significant association was found between genetic subtype and IA presence [22]. Taken together, the literature suggests that HHT1 (ENG) is more frequently associated with cerebral AVMs, spinal AVMs, and pial AVFs, while HHT2 (ACVRL1) appears more often linked to IAs. However, the overall pooled prevalence of IAs (3.3%) in our meta-analysis closely aligns with that of the general population (2–5%), which argues against a strong disease-specific association. Therefore, our findings contrast the literature by suggesting that IAs in HHT may occur sporadically and be influenced more by conventional vascular risk factors than by the underlying HHT genotype. While ACVRL1 mutations may be overrepresented in some IA cases, the current evidence does not support a clear genotype-driven mechanism for aneurysm formation in HHT.

Furthermore, Azma et al. found no clear genetic predominance (among ENG, ACVRL1, or SMAD4) in patients with DVAs or CVMs, supporting the hypothesis that these non-shunting lesions may not share the same molecular mechanisms as dAVFs and AVMs [14].

Mutations in genes involved in the TGF-β signaling pathway, such as ENG, ACVRL1, and RASA1, disrupt angiogenesis and compromise vascular integrity, contributing to abnormal vessel formation [47]. These genotype-specific patterns highlight the need for personalized screening protocols based on molecular diagnostics [48–50]. Future research should prioritize longitudinal studies in genetically stratified cohorts to refine risk assessment and improve clinical management of HHT-related cerebrovascular malformations.

### Limitations

This study has several limitations. First, the I² values exceeded 50% for many estimates, indicating substantial heterogeneity in outcome reporting across studies. A significant proportion of the included studies were retrospective single centre case series rather than prospective screening investigations, which may have led to an overestimation of non-AVM prevalence due to selection bias inherent to the patient populations assessed.

Furthermore, there was variability in the imaging modalities used to detect cerebrovascular malformations. While some studies employed high-resolution techniques such as DSA, others relied on MRI, MR angiography, or CT angiography. Notably, most HHT patients were screened with MRI alone, despite DSA being the most sensitive tool for identifying high-flow vascular lesions, potentially resulting in under-detection of certain malformations.

Despite these limitations, the study has notable strengths. We aggregated data spanning more than four decades and made a clear methodological distinction between studies evaluating unselected HHT populations and those consisting of case series, case reports, or cohorts lacking systematic screening criteria. To minimize selection bias, prevalence estimates were derived exclusively from the former, indicated with an asterisk in Table 1. While the prevalence of dAVFs in HHT patients appears higher than in the general population, this enrichment should be interpreted cautiously.

Additionally, our findings suggest a possible association, but they do not establish a definitive causal relationship. The lack of mechanistic data and the absence of prospective longitudinal studies limit our ability to determine whether dAVFs represent a true syndromic manifestation of HHT or occur independently but coincidentally in a small subset of patients, making any and conclusions purely preliminary. Therefore, our contribution should be viewed as descriptive and hypothesis-generating, providing a foundation for future work aimed at clarifying the pathophysiological relevance of dAVFs in HHT and to inform the refinement of future screening recommendations.

To our knowledge, this is the first systematic review to provide a comprehensive overview of the spectrum of non-AVM cerebrovascular malformations in HHT, offering valuable insight into their frequency, distribution, and potential genetic associations.

## Conclusion

HHT is associated with a broader cerebrovascular phenotype beyond classical AVMs. Among non-AVM malformations, only dAVFs show a significantly increased prevalence, suggesting a potential associative link. Other lesions, such as IAs, DVAs, and cavernomas, occur at rates similar to the general population. Genotype–phenotype patterns suggest ENG and ACVRL1 mutations may influence lesion type, but evidence is still limited. Standardized screening and genetic stratification are needed to refine risk assessment.

## Data Availability

Available upon reasonable request.

## Abbreviations

HHT: Hereditary Hemorrhagic Telangiectasia
AVM: Arteriovenous Malformation
IA: Intracranial Aneurysm
dAVF: Dural Arteriovenous Fistula
DVA: Developmental Venous Anomaly
CVM: Capillary Vascular Malformation
ENG: Endoglin
ACVRL1: Activin A Receptor Type II-Like Kinase 1
SMAD4: Mothers Against Decapentaplegic Homolog
